# Trends and Determinants of Prescription Drug Use during Pregnancy and Postpartum in British Columbia, 2002–2011: A Population-Based Cohort Study

**DOI:** 10.1371/journal.pone.0128312

**Published:** 2015-05-26

**Authors:** Kate Smolina, Gillian E. Hanley, Barbara Mintzes, Tim F. Oberlander, Steve Morgan

**Affiliations:** 1 Centre for Health Services and Policy Research, University of British Columbia, Vancouver, British Columbia, Canada; 2 Department of Obstetrics and Gynaecology, University of British Columbia, Vancouver, British Columbia, Canada; 3 School of Population and Public Health, University of British Columbia, Vancouver, British Columbia, Canada; 4 Child and Family Research Institute, University of British Columbia, Vancouver, British Columbia, Canada; 5 Centre for Health Services and Policy Research, University of British Columbia, Vancouver, British Columbia, Canada; Medical University of Vienna, AUSTRIA

## Abstract

**Purpose:**

To describe trends, patterns, and determinants of prescription drug use during pregnancy and postpartum.

**Methods:**

This is a retrospective, population-based study of all women who gave birth between January 2002 and 31 December 2011 in British Columbia, Canada. Study population consisted of 225,973 women who had 322,219 pregnancies. We examined administrative datasets containing person-specific information on filled prescriptions, hospitalizations, and medical services. Main outcome measures were filled prescriptions during pregnancy and postpartum. We used logistic regressions to examine associations between prescription drug use and maternal characteristics.

**Results:**

Approximately two thirds of women filled a prescription during pregnancy, increasing from 60% in 2002 to 66% in 2011. The proportion of pregnant women using medicines in all three trimesters of pregnancy increased from 20% in 2002 to 27% in 2011. Use of four or more different types of prescription drug during at least one trimester increased from 8.4% in 2002 to 11.7% in 2011. Higher BMI, smoking during pregnancy, age under 25, carrying multiples, and being diagnosed with a chronic condition all significantly increased the odds of prescription drug use during pregnancy.

**Conclusions:**

The observed increase in the number of prescriptions and number of different drugs being dispensed suggests a trend in prescribing practices with potentially important implications for mothers, their neonates, and caregivers. Monitoring of prescribing practices and further research into the safety of most commonly prescribed medications is crucial in better understanding risks and benefits to the fetus and the mother.

## Introduction

Prescribing of drugs to pregnant women has been a concern for mothers, health care providers, and the public since the thalidomide tragedy in the 1960s [1]. This is, in part, because quality information about the safety and effectiveness of medicine use during pregnancy is lacking for the majority of prescription drugs available on the market [2, 3]. Animal studies will not always predict teratogenic risks in humans and pregnant women are generally excluded from clinical trials that generate most safety and effectiveness data. Additionally, few drug information systems assessing medicine use at a population level capture data for all women of child bearing ages, limiting the amount of information generated through post-market surveillance.

There have been numerous calls for more research into prescription drug use during pregnancy and associated health risks [4, 5]. A number of studies have found that the use of prescription drugs during pregnancy is common and, though varying across jurisdictions, is increasing over time [6–10]. However, many studies published to date provide little or no examination of drug use during the postpartum period; do not report trends over time; and/or do not explore factors associated with drug use [8, 9, 11–14]. Further, information on prescribing to North American pregnant women is limited.

High-quality analyses of prescription drug use during pregnancy and lactation are needed because the availability and use of prescription medicines is changing over time. For example, several antipsychotic drugs are now approved for use in major depression and anxiety disorders [15], rates of diagnoses of ADHD have expanded significantly in adult populations [16], and there are increasing diagnoses of comorbid psychiatric conditions in pregnant populations [17]. Changing maternal characteristics are also likely to play an important role in prescription drug use in pregnancy, including the changes in mean maternal age at conception, pre-pregnancy BMI and related maternal conditions [18].

For these reasons, research on the use of prescription medicines in pregnancy and the postpartum period remains critically important. Defining how commonly medicines are used during pregnancy and postpartum, which medicines are most often used, and how patterns of such use are changing over time will help define new research priorities in this field. This study describes the trends, patterns, and determinants of prescription drug use during pregnancy and postpartum among women living in the Canadian province of British Columbia (BC).

## Methods

### Data Sources

Our analysis is based on de-identified linked health datasets provided by Population Data BC with approval of relevant data stewards and of the University of British Columbia’s Behavioural Research Ethics Board (H10-01002) [19–21]. Information about maternal and infant health came from the Perinatal Services BC’s Perinatal Data Registry (PDR). This province-wide database includes information on antenatal, intrapartum/delivery and postpartum maternal and infant care and outcomes for nearly all births in British Columbia (approximately 99%). Some information on maternal reproductive history is also recorded (e.g. previous miscarriages, stillbirths, and premature deliveries), including information on maternal smoking during pregnancy.

The PDR data was linked to information about women’s prescription drug dispensations, medical services use, hospitalizations, and income, as well as vital statistics for their babies. Information about prescription dispensations—including drug type and quantity—came from BC PharmaNet, an information system that records every prescription filled outside of acute care hospitals in British Columbia, regardless of patient age or insurance status. Medical and hospital data were obtained from British Columbia’s universal, public health insurance program. Income quintiles were estimated using government records of the average adjusted household income in each patient’s neighborhood, with neighborhoods including approximately 400–700 residents [22].

### Study Population

We identified all women in the BCPDR database who gave birth between 1 January 2002 and 31 December 2011. To ensure complete capture of relevant prescription dispensations and health information for all women in our cohort, we excluded women who were not registered for the universal public health insurance program (and therefore likely not residing in the province) for at least 275 days in each year from one year prior to pregnancy to one year following pregnancy. The public health insurance plan covers all permanent residents of BC with the exception of approximately 4% of the population who is covered by various federal health insurance programs.

We used the Aggregated Diagnosis Groups of the Johns Hopkins Adjusted Clinical Groups (ACG) Case-Mix System (version 10.0) to identify women with chronic conditions based on diagnoses recorded in hospital and physician billing records. The Appendix provides information on which diagnostic groups were used to identify women who have a chronic disease. The ACG case-mix system has been shown to be predictive of both drug use and expenditure in the BC population [23].

### Defining the pregnancy period

Vital Statistics data provided exact dates of birth for all babies born to the women in our cohort. We also had access to highly reliable gestational age estimates based on information from early-gestation ultrasound and/or the date of last menstrual period (available for more than 90% of records). If neither field was recorded, gestational age was estimated from a newborn clinical exam and/or chart documentation. Information on final estimate of gestational age was available for 99% of all records.

We estimated the date of conception using the following formula: (birth date—gestational age in weeks * 7) + 14 days. We then defined the following eight pregnancy-related periods: one prepartum period up to three months before pregnancy; three pregnancy periods, including first trimester (0–90 days), second trimester (91–181 days), and third trimester (182 days to delivery); and four postpartum periods, including up to three months, four to six months, seven to nine months, and 10 to 12 months after delivery.

### Prescription Drug Information

We used the World Health Organization Anatomical Therapeutic Chemical (ATC) drug classification system to distinguish drug classes (third level of the ATC system, pharmacological subgroups) and drug types (fifth level of the ATC system, chemical substances) [24]. Our measure of exposure to a medicine during pregnancy and specific pregnancy-related periods is based on the date the prescription was filled and thus drug dispensed to the patient.

While the majority of pregnant women purchase their vitamins and minerals over the counter, we included the vitamins that were obtained via prescription in this study, as women who obtain their vitamins and minerals via prescription may do so for different indications, including those that require higher dosage. However, given that the data on use of vitamins and minerals are incomplete, and that use of supplements is often considered differently from other prescription medicine use, we also present results that do not include vitamins and minerals.

### Statistical Analyses

We present descriptive statistics on the prevalence of drug use during pregnancy-related periods. We used chi-square tests to test for time trends from 2002 to 2011. We used univariable and multivariable logistic regression to generate crude and adjusted odds ratios (ORs) and 95% confidence intervals (CIs) for health, demographic, and socio-economic maternal characteristics associated with prescription drug use during pregnancy.

As part of sensitivity analyses, we excluded women who filled only one prescription during the first two months of pregnancy, as these may represent prescriptions filled prior to knowledge of the pregnancy and not used once the woman became aware she was pregnant. As a woman’s subsequent pregnancies are unlikely to be independent, in terms of determinants of medicine use, we also ran models including only the first pregnancy for each woman to explore effects on outcomes. Finally, we ran models excluding any dispensations of prescription vitamins and minerals. All analyses were performed using Stata version 12.1 (College Station, TX) and SAS version 9.3 (SAS Institute, Cary, NC).

This study was approved by the University of British Columbia’s Behavioural Research Ethics Board (H10-01002; April 19, 2010).

## Results

We identified 225,973 women who had 322,219 pregnancies between 1 January 2002 and 31 December 2011 in British Columbia. Table 1 provides maternal characteristics of the study cohort. Almost two thirds of women (64%) had only one pregnancy during the study period. Mean age at delivery was 30.9 (range 11–56 years) overall, increasing from 30.7 in 2002 to 31.2 in 2011. Prescriptions were dispensed to women during 206,680 of the pregnancies (64%) in our study. With prescriptions for vitamins and minerals excluded, this number fell to 200,636 pregnancies (62%).

**Table 1:** Maternal characteristics of the study cohort.

	All pregnancies, total n	Did not fill any prescriptions during pregnancy, %	Filled ≥1 prescriptions during pregnancy, %	Crude OR	95% CI
TOTAL pregnancies	322,219	115,539	206,680		
**Age (years)**					
<25	41,134	12	13	1.22	1.19–1.24
25–29	83,929	26	26	1.08	1.06–1.10
30–34	111,439	36	34	Ref.	
35–39	68,526	22	21	1.04	1.02–1.06
40+	17,191	5.1	5.5	1.15	1.11–1.19
Mean (SD)	30.9(5.4)	30.9(5.5)	31(5.3)		
**Pre-pregnancy BMI**					
<18.5 (underweight)	12,405	4.0	3.8	1.05	1.01–1.09
18.5–24.9 (normal)	146,418	49	43	Ref.	
25.0–29.9 (overweight)	50,364	15	16	1.24	1.21–1.26
30+ (obese)	28,627	7.0	10	1.60	1.56–1.65
Unknown	84,405	25	27		
**Smoked during pregnancy**					
No	293,341	92	90	Ref.	
Yes	28,878	7.5	10	1.33	1.30–1.37
**Parity**					
0	143,114	46	44	Ref.	
1+	179,105	54	56	1.07	1.06–1.09
**Pregnancy type**					
Singleton	316,978	99	98	Ref.	
Multiples	5,241	1.1	1.9	1.74	1.63–1.85
**Gestation (weeks)**					
<20	191	0.1	0.1	0.71	0.53–0.94
20–36 (preterm)	27,675	7.3	9.3	1.29	1.25–1.32
37+ (term)	293,936	92	91	Ref.	
Unknown	417	0.4	0.0		
Mean (SD)	38.6(2.2)	38.6(2.2)	38.8(2.2)		
**Income quintile**					
Lowest	64,838	18	21	1.23	1.20–1.26
2nd	64,352	20	20	1.09	1.06–1.11
3rd	64,229	20	20	1.03	1.00–1.05
4th	64,374	21	20	1.02	1.00–1.05
Highest	64,177	21	19	Ref.	
Unknown	249	0.05	0.09		
**Chronic disease**					
No	192,699	71	54	Ref.	
Yes	129,520	29	46	2.11	2.08–2.14
**Reproductive history**					
Low birth weight	6,156	1.5	2.1	1.38	1.30–1.46
Stillbirth	2,491	0.7	0.8	1.24	1.14–1.35
Preterm	12,359	3.2	4.2	1.30	1.25–1.35
Spontaneous abortion	71,364	20	23	1.17	1.15–1.19
Neonatal death	1141	0.3	0.4	1.33	1.17–1.51
Congenital anomaly	2,434	0.7	0.8	1.16	1.06–1.20

BMI = body mass index; CI = confidence interval; OR = odds ratio; Ref. = reference; SD = standard deviation

**Fig 1:**
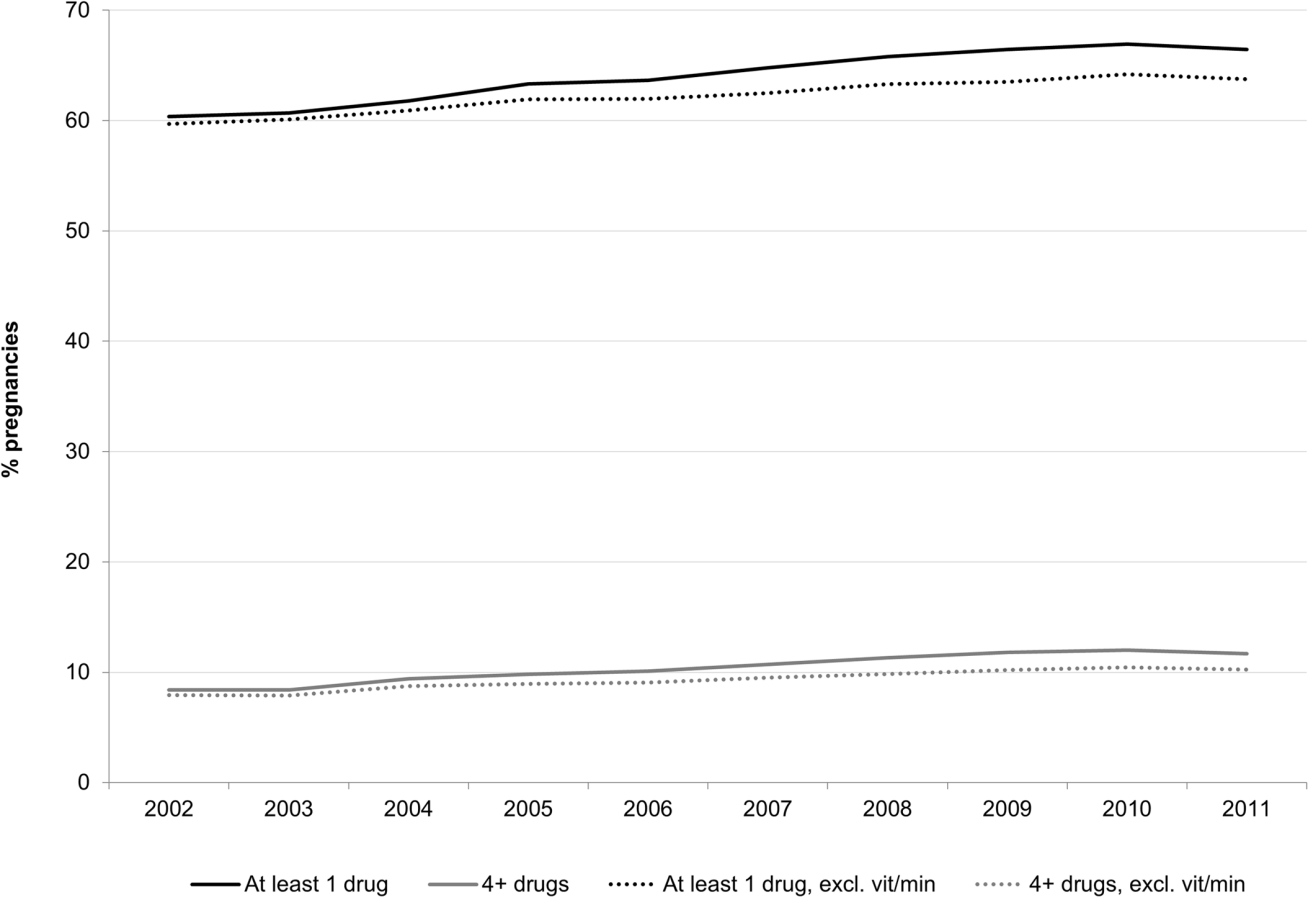
Overall patterns of prescription drug dispensations during pregnancy.

As illustrated in Fig 1, the proportion of pregnancies involving at least one prescription drug dispensation increased from 60% in 2002 to 66% in 2011 (p<0.001). Excluding vitamins and minerals, the change was 60% to 64% (p<0.001).Prevalence of prescription drug use increased among all age groups.

The mean and median number of dispensations among women who had at least one dispensation during pregnancy were 4.5 and 3, increasing from 3.9 and 2 in 2002 to 5.3 and 3 in 2011, respectively. Excluding vitamins and minerals, the overall mean and medians were 4.3 and 2, increasing from 3.9 and 2 in 2002 to 5.1 and 3 in 2011. However, because methadone and suboxone are daily dispensations, they greatly influence the average figures. Excluding these two drugs reduced the mean number of dispensations to 3.6 in 2002 and 4.7 in 2011.

Increases in the average number of dispensations were observed in every age group. The mean number of dispensations was similar for first and subsequent pregnancies. Older women were dispensed more drugs—those 40 years and older had an average of 5.0 dispensations per pregnancy, while those 25 years and younger had an average of 4.2 dispensations.

**Fig 2:**
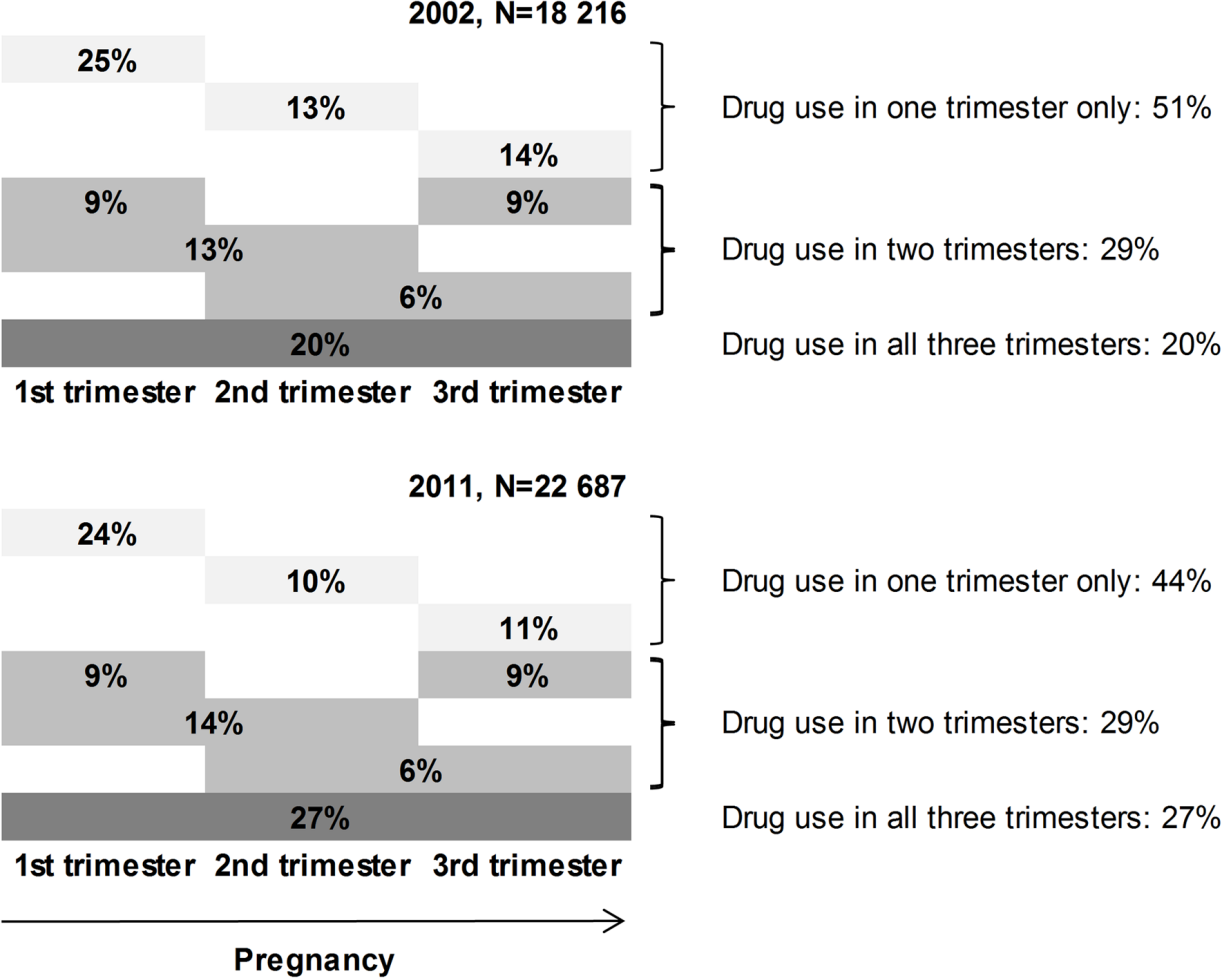
Trimesters during which prescriptions were dispensed to women during pregnancies involving at least one prescription dispensation (number of pregnancies).

As Fig 2 illustrates, the distribution of dispensations across the pregnancy period changed over the study period: compared to 2002, a smaller proportion of women received a prescription drug during only one trimester in 2011 (44% vs 51%) and a higher proportion of women received drugs during all three trimesters (27% vs 20%). Exclusion of vitamins and minerals generated similar proportions and changes over time.

**Fig 3:**
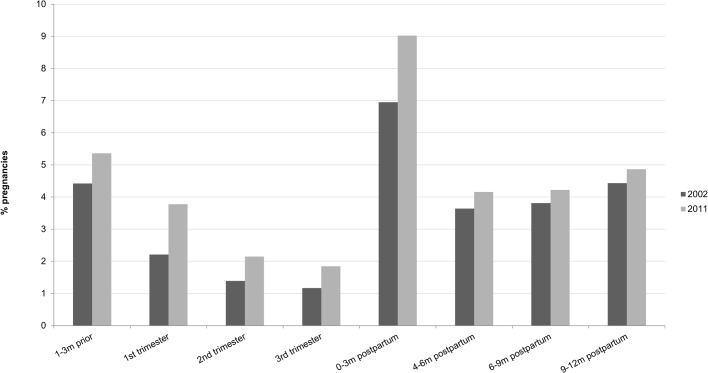
Pregnancies involving dispensation for four or more different drug types, by time period and year (excluding vitamins and minerals).

The proportion of pregnancies for which four or more different prescription drug types were dispensed during one of the three trimesters increased from 8.4% in 2002 to 11.7% in 2011 (see Fig 1). Excluding vitamins and minerals, the change was 7.9% to 10.3%. As shown in Fig 3, increases in the proportion of women who were dispensed four or more different drugs were observed for all pregnancy-related periods. Results excluding vitamins and minerals were similar.

**Fig 4:**
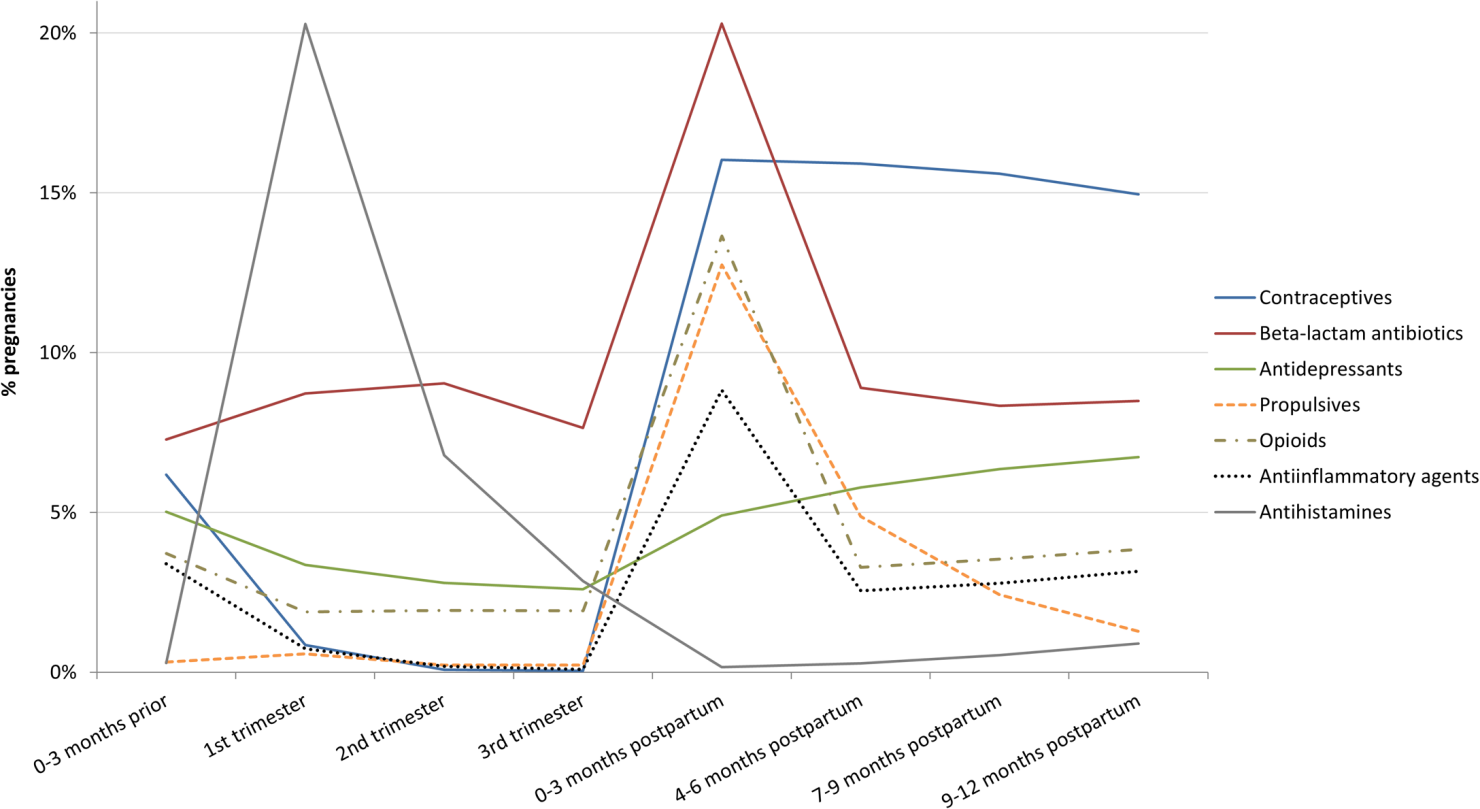
Patterns of prescription drug dispensations for most common drug classes by pregnancy-related period.

Table 2 details all and top five most commonly dispensed drugs during the three months pre-pregnancy, in each trimester of pregnancy, and in each three-month period for the first year postpartum. Overall, the most commonly prescribed medication was doxylamine-pyridoxine HCL (brand-name Doxylamine-pyridoxine in Canada, an antiemetic; a similar formulation was called Bendectin in the US) among all age groups, closely followed by amoxicillin (an antibiotic). Fig 4 illustrates the percentage of pregnancies exposed to select drug classes by three-month period prior to, during, and after pregnancy.

**Table 2:** Number and type of prescription drugs dispensed by trimester and pre- and post-pregnancy period: number of pregnancies with at least one prescription (percent of all pregnancies).

Drug	0–3 months prior n (%)	1^st^ trimester n (%)	2^nd^ trimester n (%)	3^rd^ trimester n (%)	Pregnancy n (%)	0–3 months postpartum n (%)	4–6 months postpartum n (%)
All drugs; N = 322,219	129,909 (40)	145,359 (45)	113,492 (35)	107,278 (33)	206,680 (64)	204,306 (63)	150,058 (47)
Excl. vitamins	128,616 (40)	138,365 (43)	106,405 (33)	101,464 (31)	200,636 (62)	203,274 (63)	149,176 (46)
4+ drugs; N = 322,219	15,460 (4.8)	9,502 (2.9)	5,944 (1.8)	5,111 (1.6)	33,592 (10)	27,635 (8.6)	12,729 (4.0)
Excl. vitamins	15,059 (4.7)	8,049 (2.5)	5,262 (1.6)	4,584 (1.4)	30,087 (9.3)	26,848 (8.3)	12,489 (3.9)
Top 5 most common drugs, 2002; N = 30,167	Amoxicillin 1,203 (4.0)	Doxylamine5,026 (17)	Amoxicillin 1,922 (6.4)	Amoxicillin 1,740 (5.8)	Doxylamine5,337 (18)	Codeine 4,369 (14)	Norethisterone1,679 (5.6)
	Codeine 1,150 (3.8)	Amoxicillin 1,758 (5.8)	Doxylamine 1,692 (5.6)	Insulin 978 (3.2)	Amoxicillin4,776 (16)	Cefalexin3,085 (10)	Amoxicillin1,331 (4.4)
	Salbutamol 598 (2.0)	Levothyroxine 669 (2.2)	Levothyroxine 715 (2.4)	Hydrocortisone cream 947 (3.1)	Hydrocortisone cream1,888 (6.3)	Domperidone2,259 (7.5)	Levonogestrel1,285 (4.3)
	Levonorgestrel 589 (2.0)	Salbutamol 584 (1.9)	Hydrocortisone cream 657 (2.2)	Doxylamine646 (2.1)	Codeine 1,366 (4.5)	Norethisterone2,257 (7.5)	Codeine1,025 (3.4)
	Levothyroxine 585 (1.9)	Codeine 525 (1.7)	Salbutamol 596 (2.0)	Levothyroxine 616 (2.0)	Salbutamol 1,219 (4.0)	Hydrocortisone cream1,869 (6.2)	Medroxyprogesterone817 (2.7)
Top 5 most common drugs, 2011; N = 34,148	Amoxicillin 1,359 (4.0)	Doxylamine7,731 (23)	Doxylamine 2,456 (7.2)	Amoxicillin 1,639 (4.8)	Doxylamine8,078 (24)	Domperidone6,014 (18)	Domperidone2,401 (7.0)
	Codeine 1,074 (3.1)	Amoxicillin 2,051 (6.0)	Amoxicillin2,047 (6.0)	Ranitidine 1,166 (3.4)	Amoxicillin5,080 (15)	Cefalexin3,543 (10)	Norethisterone2,394 (7.0)
	Levothyroxine 903 (2.6)	Iron2,040 (6.0)	Iron1,490 (4.4)	Levothyroxine1,136 (3.3)	Iron2,437 (7.1)	Norethisterone3,206 (9.4)	Amoxicillin1,229 (3.6)
	Clomifene681 (2.0)	Levothyroxine 669 (2.2)	Levothyroxine1,325 (3.9)	Doxylamine 1,087 (3.2)	Ranitidine1,919 (5.6)	Diclofenac2,178 (8.0)	Levothyroxine 1,116 (3.3)
	Ciprofloxacin632 (1.9)	Folic acid844 (2.5)	Ranitidine 855 (2.5)	Iron1,071 (3.1)	Hydrocortisone cream1,842 (5.4)	Codeine2,608 (7.6)	Levonorgestrel874 (2.6)

**Fig 5:**
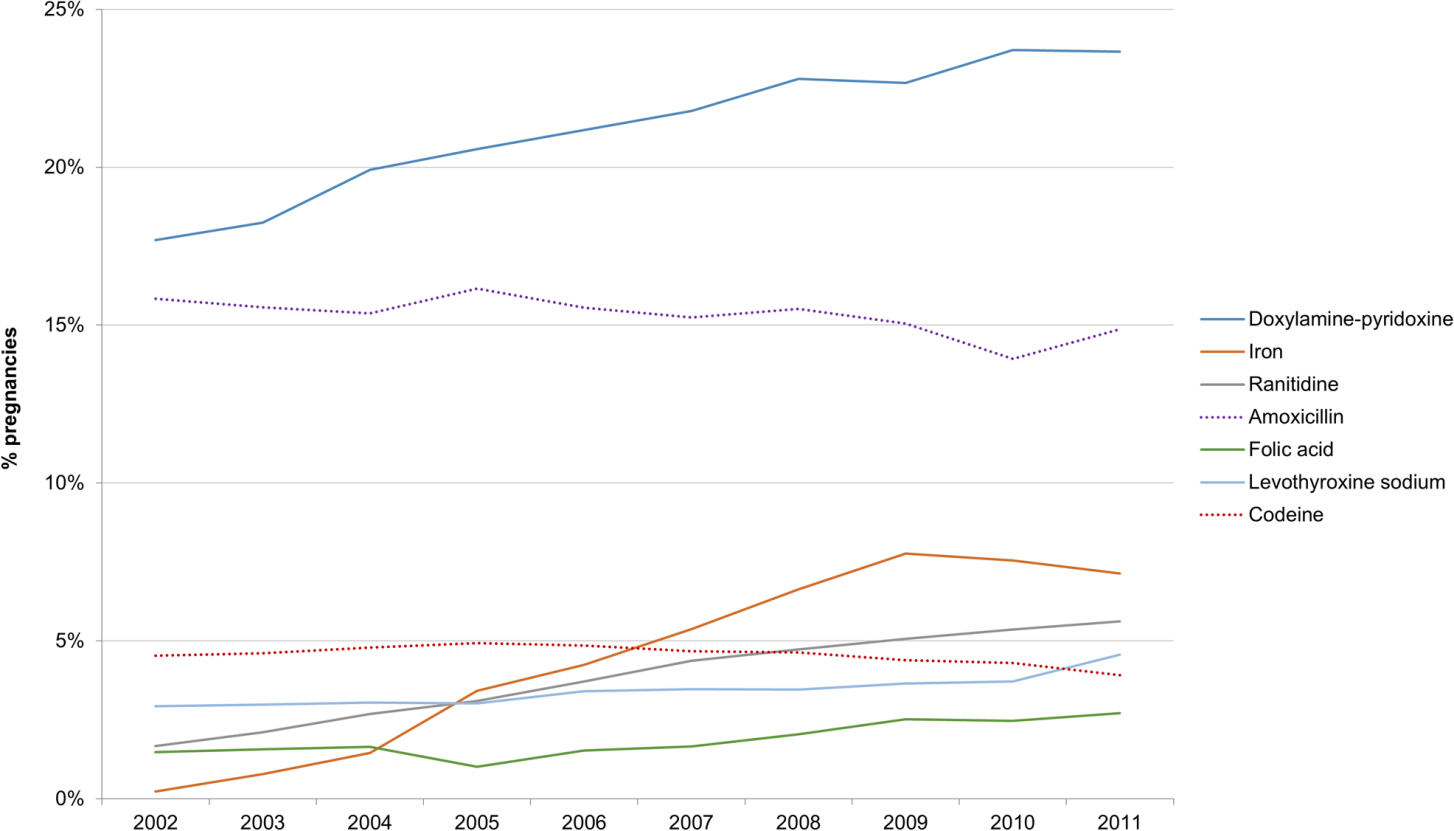
Trends in prescription drug dispensations during pregnancy, most common drugs, 2002–11.

Over the study period, the proportion of pregnancies in which women were dispensed prescriptions for antibiotics, opioids, antidepressants, and anxiolytics did not change to any great extent (data not shown). Correlating with a Health Canada safety letter sent to physicians in 2005,[25] the rate of paroxetine prescribing fell between 2005 and 2007. As Fig 5 illustrates, the use of doxylamine-pyridoxine and PregVit iron supplements during pregnancy increased significantly over the study period (p<0.001 for both). Despite doxylamine-pyridoxine being responsible for a large share of all prescriptions to pregnant women, a temporal increase in the use of prescription drugs during pregnancy was observed even after excluding doxylamine-pyridoxine.

Women who were pregnant with multiples (ORa = 1.79, CI: 1.66–1.93), were under age 25 (ORa = 1.19, CI: 1.16–1.23), had a BMI of 30 or higher (ORa = 1.46 CI: 1.42–1.51), who smoked during pregnancy (ORa = 1.17, CI: 1.13–1.21), were in the lowest income quintile (ORa = 1.15, CI: 1.12–1.19), and had a chronic disease (ORa = 2.01, CI: 1.98–2.05) were more likely to fill a prescription during pregnancy (Table 3). These results did not substantially change after sensitivity analyses looking at women with first pregnancies only; after excluding vitamins and minerals; or after excluding women with only one dispensation in the first two months of pregnancy (data not shown).

**Table 3:** Factors associated with prescription drug use during pregnancy.

	All pregnancies					
	All		With chronic disease		Without chronic disease	
	N = 236,066		N = 94,778		N = 141,288	
	ORa	95% CI	ORa	95% CI	ORa	95% CI
**Age (years)**						
<25	**1.19**	(1.16–1.23)	**1.17**	(1.11–1.23)	**1.21**	(1.16–1.26)
25–29	**1.06**	(1.04–1.09)	**1.08**	(1.04–1.12)	**1.06**	(1.03–1.09)
30–34	**Ref.**		**Ref.**		**Ref.**	
35–39	**1.02**	(1.00–1.05)	**1.04**	(1.00–1.08)	**1.02**	(0.99–1.05)
40+	**1.09**	(1.05–1.14)	**1.16**	(1.09–1.24)	**1.05**	(0.99–1.10)
**BMI**						
<18.5 (underweight)	**1.02**	(0.98–1.06)	**0.96**	(0.90–1.03)	**1.06**	(1.01–1.11)
18.5–24.9 (normal)	**Ref.**		**Ref.**		**Ref.**	
25.0–29.9 (overweight)	**1.19**	(1.16–1.22)	**1.27**	(1.22–1.32)	**1.15**	(1.12–1.18)
30+ (obese)	**1.46**	(1.42–1.51)	**1.60**	(1.53–1.68)	**1.38**	(1.33–1.43)
**Smoked during pregnancy**						
No	**Ref.**		**Ref.**		**Ref.**	
Yes	**1.17**	(1.13–1.21)	**1.30**	(1.23–1.37)	**1.10**	(1.05–1.14)
**Pregnancy type**						
Singleton	**Ref.**		**Ref.**		**Ref.**	
Multiples	**1.79**	(1.66–1.93)	**1.71**	(1.50–1.94)	**1.84**	(1.67–2.02)
**Reproductive history**						
Low birth weight	**1.21**	(1.12–1.30)	**1.09**	(0.97–1.23)	**1.29**	(1.17–1.41)
Preterm	**1.15**	(1.09–1.22)	**1.27**	(1.17–1.39)	**1.08**	(1.01–1.15)
Spontaneous abortion	**1.12**	(1.10–1.15)	**1.14**	(1.10–1.18)	**1.11**	(1.08–1.15)
**Parity**						
0	**Ref.**		**Ref.**		**Ref.**	
1	**1.05**	(1.04–1.07)	**1.12**	(1.09–1.16)	**1.02**	(1.00–1.04)
**Income quintile**						
Lowest	**1.15**	(1.12–1.19)	**1.22**	(1.17–1.28)	**1.11**	(1.07–1.15)
2nd	**1.05**	(1.02–1.08)	**1.08**	(1.03–1.13)	**1.03**	(0.99–1.06)
3rd	**0.99**	(0.96–1.02)	**1.04**	(0.99–1.09)	**0.96**	(0.93–0.99)
4th	**0.99**	(0.96–1.02)	**1.03**	(0.98–1.07)	**0.97**	(0.94–1.00)
Highest	**Ref.**		**Ref.**		**Ref.**	
**Chronic disease**						
No	**Ref.**					
Yes	**2.01**	(1.98–2.05)				

## Discussion

### Main findings

In this analysis of 322,219 pregnancies in the population of British Columbia, Canada, we found that at least one prescription was filled during pregnancy by nearly two thirds of women. Between 2002 and 2011, there was a 10% increase in the proportion of pregnant women filling a prescription as well as the number of prescriptions and number of different drugs among those who fill at least one prescription. Factors significantly associated with use of prescription drugs during pregnancy included high BMI, smoking, age under 25, carrying multiple fetuses, and having one or more chronic diseases, among others.

Among notable findings from our study, we found that more than one in ten pregnant women in 2011 used four or more different medicines during pregnancy. This represents a 28% increase over the rate of exposure to four or more medicines in 2002 (8.4%), a finding that has important safety implications. While for many drugs in our study, little research has been carried out on effects on the fetus, we have even less information on the effects of combinations of medications during pregnancy. There is some evidence that exposure to more than one class of psychotropic medicines may increase health risks to the infant. Oberlander et al. found higher risks of congenital heart defects among infants whose mothers were using both SSRI antidepressants and benzodiazepines during pregnancy [26]. There are also important research-related implications of this finding. Most research on prescription drug use during pregnancy examines a single therapeutic class and attempts to control for confounding by the underlying indication for the medicine. Our study suggests that it is relatively common for women to be treating multiple conditions with multiple medications during pregnancy.

### Interpretation

Our estimates of the prevalence of prescription drug use and mean number of prescriptions dispensed during pregnancy are generally in agreement with the rates reported in the literature for the years included in the study period [8–11, 27]. However, the reported rates range from 28% to 93% [13, 14, 28–31]—a reflection of differences in both study design and jurisdictional differences in prescribing practices.

Among studies reporting prevalence of drug use by trimester, trimester-specific rates of use differ: some studies have reported an increase between the first and third trimester [14, 29, 32], while others report minimal changes [8, 11, 33, 34] or a decline [27]. We found that the rate of use decreased beyond the first trimester, consistent with a decline in morning sickness. Further, some of the first trimester exposures occur before the woman is aware she is pregnant.

We also found different patterns of prescription drug dispensations across pregnancy-related periods compared to other studies that reported drug class-specific rates of use before, during, and after pregnancy [8, 11, 12, 29, 33]. That was expected, given that other studies were also not consistent with each other, reflecting regional differences in prescribing practices. In British Columbia, doxylamine-pyridoxine and amoxicillin are the two most commonly dispensed medications during pregnancy. This is in line with the biological course of pregnancy, with nausea/vomiting and frequent urinary tract infections being two common pregnancy-related conditions. Levothyroxine is prescribed for hypothyroidism, which is often screened for in pregnancy and treated more aggressively than in the non-pregnant population, with treatment also recommended for subclinical hypothyroidism. Domperidone, a propulsive indicated for functional gastrointestinal disorders, has emerged over the last decade as the standard (off-label) treatment in Canada for postpartum women with lactation difficulties. Codeine use postpartum has decreased, in light of the FDA and Health Canada advisories in 2007 and 2008, respectively, warning about the potentially life-threatening adverse effects in babies of breastfeeding mothers taking codeine.[35, 36] The increased use of iron during pregnancy over the study period is driven by the introduction of a new prescription vitamins and minerals supplement tablets (Pregvit) to the Canadian market in 2003.

Our findings of a rapid increase in doxylamine-pyridoxine use during pregnancy between 2002 and 2011 are consistent with increased prescribing in seven other Canadian provinces [37]. Since 2002, Canadian guidelines have recommended doxylamine-pyridoxine first-line use for nausea and vomiting in pregnancy [38]. Effectiveness is mild: the only placebo-controlled trial of the Canadian formulation (Diclectin), published in 2010 (n = 256), found a mean 0.7-point difference (95% CI 0.2 to 1.3) versus placebo on a 13-point symptom scale [39]. Doxylamine-pyridoxine was withdrawn from the US market in 1983 following lawsuits over potentially drug-related birth defects, although a 1999 US FDA review judged that withdrawal was “…for reasons other than safety or effectiveness”[40]. Diclectin, introduced in 2013, is the first doxylamine-pyridoxine product in the US since 1983. Observational studies indicate no increase in total malformations[37] but a protective effect widely cited in Canada was erroneous [41], and ongoing questions remain about specific risks, such as pyloric stenosis (narrowing of the passage from the stomach to the intestine) [37].

Our results for factors associated with drug use in pregnancy are consistent with the published literature.[42–45] In a Canadian study that explored maternal characteristics associated with exposure to harmful medications (defined as FDA category C, D, or X drugs), age younger than 25 (ORa = 1.2), parity of three or more (ORa = 1.25) and lower income (ORa = 1.93) were found to be statistically significant [42]. Another study of over 61,000 Irish women found similar adjusted ORs for the use of any medication during pregnancy for smoking (ORa = 1.12), carrying multiples (ORa = 1.28), and age of 40 or over (ORa = 1.11) [43]. A US study found chronic disease and parity of one or more to be associated with exposure to FDA category D and X drugs [44]. A recent cross-sectional, multinational web-based study identified older age, previous children, and smoking to be associated with medication use in pregnancy, among other factors [45].

### Strengths and Limitations

Our findings are strengthened by the prospective collection of the data we analysed, the inclusion of the entire population of pregnant women in BC, and the comprehensiveness of our prescription drug data used, as PharmaNet captures all drug dispensations in retail pharmacies, irrespective of the insurance status or level of coverage. We also benefit from high quality data on gestational age and birth certificate information that allowed use to calculate the most accurate exposure period for the pregnancy available for retrospective studies of administrative data.

However, our study is not without limitations. We did not examine over-the-counter medications or drugs dispensed in hospital. It is possible that this led to an underestimation of prevalence of use, particularly for drugs that are commonly available over the counter (e.g. ranitidine) or usually administered by infusion in a hospital setting (e.g. rheumatic drugs). Importantly, this study used data on drugs dispensed, which is not the same as drugs used. However, while not equivalent to a measure of consumption by a patient, dispensation information is a more accurate measure of drug exposure than data on prescriptions written because, for a variety of reasons, many prescriptions written by doctors are not filled by patients [46]. Further, to account for potential overestimation of consumption, we provide conservative estimates that remove women who filled only one prescription in the first two months of pregnancy, as these women seem most likely to have filled prescriptions that might not have been used. We were also unable to identify pregnancies that ended in miscarriage or termination, a population which may have higher exposure to medicines [47]. Any bias created through these omissions is likely to be in the direction of underestimating exposure. Data on other socioeconomic indicators (e.g. years of education, lone parenthood) that were previously shown to be associated with drug use in pregnancy [43, 44, 48] was limited and thus it was not possible to account for these factors.

## Conclusion

This population-based study shows that the majority of Canadian women use prescription medications during pregnancy and postpartum. The observed increase over the last decade in the number of prescriptions and number of different drugs being dispensed across all three trimesters and all age groups suggests a general shift in prescribing practices to pregnant women. Additionally, we noted several important drug-specific trends, particularly for doxylamine-pyridoxine in pregnancy and domperidone in the postpartum period. An increasing number of women who are taking prescription medicines in all three trimesters suggests that the way medicines are being used during pregnancy is changing, with potentially important implications for mothers, their neonates, and caregivers. Monitoring of prescribing practices and further research into the safety of most commonly prescribed medications is crucial in better understanding risks and benefits to the fetus and the mother.
